# A Study of Response Inhibition in Overweight/Obesity People Based on Event-Related Potential

**DOI:** 10.3389/fpsyg.2022.826648

**Published:** 2022-03-03

**Authors:** Ze-Nan Liu, Jing-Yi Jiang, Tai-Sheng Cai, Dai-Lin Zhang

**Affiliations:** The Medical Psychological Institute, The Second Xiangya Hospital of Central South University, Changsha, China

**Keywords:** overweight, obesity, response inhibition, event-related potential, Go/Nogo, stop-signal test

## Abstract

**Objective:**

To investigate the characteristics of response inhibition of overweight/obese people, using behavior experiments combine with neural electrophysiological technology and discussing the difference in impulse level between obesity/overweight and normal-weight people through EEG data, questionnaire, and behavior experiment.

**Method:**

(1) All participants completed the Go/Nogo task; meanwhile, behavior data and 64 channel EEG data were recorded. (2) Participants completed the Stop-Signal task and behavior date was recorded.

**Results:**

(1) During Go/Nogo task, no significant differences were found in reaction time, omission errors of the Go task between the two groups, while commission errors of the Nogo task of the control group were significantly greater than the overweight/obesity group. (2) About SSRT during the Stop-Signal Task, the interaction of stimulus type (high-calorie food picture, low-calorie food picture) and group (control group, overweight/obesity group) was significant (*p* = 0.008). (3) No significant differences were found between the two groups in amplitude and latency of N2. About the amplitude of P3, the interaction of task type (Go task, Nogo task), electrode point (Cz, CPz, Pz), and groups were significant (*p* = 0.041), the control group P3 amplitude was significantly greater than overweight/obesity group during the Nogo task. Regarding about latency of P3, the interaction of group and electrode point were not significant (*p* = 0.582), but the main effect of task type was significant (*p* = 0.002).

**Conclusion:**

(1) In terms of behavioral outcomes, overweight–obese subjects had lower dominant response inhibition and response cessation compared to normal-weight subjects. (2) In terms of EEG results, overweight–obese subjects showed no difference in processing speed and level of conflict monitoring for early inhibitory processing compared to normal-weight subjects, but there was a deficit in behavioral control for late inhibitory processing.

## Introduction

Obesity/overweight is a chronic disease caused by excessive energy intake by the body, resulting in the accumulation of fat or excessive weight beyond the normal range. WHO defines obesity/overweight with body mass index (BMI). BMI in the range of 25 ~ 29.9 kg/m^2^ as overweight and BMI ≥ 30 kg/m^2^ as obese (Deitel and Greenstein, 2003). In China, the latest epidemiological survey in 2020 showed that the overweight and obesity rates of residents aged 18 years and above were 34.3 and 16.4%, respectively. And overweight and obesity rates among children and adolescents have increased to varying degrees (Liu, 2020). The problem of obesity and overweight is becoming increasingly prominent. Existing studies have found that higher cancer mortality in obese individuals (Calle et al., 2003), and other diseases have shown that high levels of BMI are associated with high mortality (Flegal et al., 2018). Maternal obesity can harm the learning, motivation, and neuro-evolution of their offspring (Contu et al., 2017). Also, obesity or overweight can cause other chronic diseases, such as diabetes and heart disease (Quek et al., 2017), in addition to impairing the individual’s inhibitory control, attention, and memory (Yang et al., 2018; Mora-Gonzalez et al., 2019; Türkoğlu and Çetin, 2019). Overweight/obese individuals are significantly impaired particularly in executive function. Executive function is a complex system in which individuals coordinate multiple cognitive subcomponents during the completion of goal-directed behaviors, mainly including working memory, cognitive switching, and inhibitory control (Smith and Jonides, 1999). Inhibitory control, also called self-control, is a central component of executive function. It can reflect inhibition at the behavioral level and is the control of an individual’s exercise over their attention, behavior, thoughts, and emotion (Smith and Jonides, 1999; Zelazo and Carlson, 2012; Diamond, 2013). What’s more, inhibition control increases with age (Luna, 2009). Inhibitory control can be divided into response inhibition and interference inhibition (Diamond, 2013). Response inhibition (RI) is the ability to inhibit behaviors that do not meet current or inappropriate needs, which is also known as self-control. RI includes both avoiding impulsive responses and resisting temptations (Hasselbalch et al., 2011; Yi et al., 2015). For obese or overweight individuals, deficits in response inhibition are mainly manifested by the inability to suppress cravings for high-calorie foods and frequent failures in the execution of weight loss plans (Blume et al., 2018). It has also been shown that obese adolescent girls have significantly lower response inhibition to food than normal-weight girls of the same age (Batterink et al., 2010).

An event-related potential (ERP) is now commonly used to study response inhibition. ERP is a potential generated by an external stimulus acting on the sensory system or on a part of the brain that causes the brain to process information about the stimulus event. The main paradigm of ERP on response inhibition in obese/overweight individuals include two types: the first type is the rapid decision experiment paradigm, such as the Stroop task, Oddball task, Go/Nogo task, and Stop-Signal test (SST). These tasks all involve direct measures of inhibition at the behavioral level and can be used to assess inhibition implementation effects. The second type is the reward setting paradigm, which is used to assess the motivational effects of inhibition. Including the Door Opening Task (DOT), the Delayed Gratification Task, and the Delayed Discount Task. These two types, respectively, correspond to impulse avoidance and temptation resistance aspects of response inhibition (Dougherty et al., 2005).

The Go/Nogo paradigm is recognized as the classical task for response inhibition measurement, which focuses on the wave amplitude and latency length of both N2 and P3 components (Smith et al., 2007; Yin, 2016). A study of obese individuals found that obese individuals’ wave amplitudes are smaller (Song et al., 2016); obese children showed a smaller range of head EEG topography activation in the Go/Nogo task, and childhood obesity is negatively correlated with inhibitory control and action monitoring (Keita, 2015); another study of obese children found smaller wave amplitudes of error-related negative waves in obese children (Skoranski et al., 2013). From a conflict information processing perspective, response inhibition can be understood as an individual’s conflict monitoring and conflict resolution of target enticement. When conflict processing was studied with Go/Nogo task, it was found that Nogo stimuli elicited significant N2 and P3 components compared to Go stimuli (Falkenstein et al., 1999; Bokura et al., 2001). And Dimoska further demonstrated with the Stop-Signal task that Nogo-N2 is associated with conflict monitoring or response selection, while Nogo-P3 is a physiological indicator of response inhibition (Dimoska et al., 2006). According to previous studies, Nogo-N2 is a negative wave that appears in the frontal–central region 200–300 ms after stimulation, and it is mainly associated with conflict monitoring (Enriquez-Geppert et al., 2010). As for Nogo-P3, it is a positive wave that appears in the central–parietal region 300–500 ms after stimulation, indicating the completion of inhibitory processing (Falkenstein et al., 1999). Therefore, Nogo-N2 and Nogo-P3 are commonly used as measures of the two phases of response inhibition. A study of obese/overweight restrictive dieters found that the subjects with better levels of conflict monitoring had higher N2 wave amplitudes and tended to choose low-calorie foods rather than high-calorie foods (Zhang, 2016). All of these studies suggest a decrease in overweight or obese individuals’ response monitoring ability.

Existing studies have found that response inhibition involves the right hemispheric response inhibition network (RIN), which includes the right inferior frontal gyrus (rIFG), the pre-supplementary motor area (preSMA), the right middle frontal gyrus (rMFG), the dorsolateral prefrontal cortex, and the anterior cingulate cortex (ACC), motor area (preSMA), right middle frontal gyrus (rMFG), dorsolateral prefrontal cortex, anterior cingulate cortex (ACC), inferior parietal lobule, insula, and subcortical structures (thalamus, caudate nucleus), etc. (Aron, 2011; Bari and Robbins, 2013).

Studies of response inhibition in individuals of different ages have revealed that segregation of executive functions in the prefrontal cortex occurs during development: activation of the left inferior frontal gyrus/insula/orbitofrontal gyrus increases with age, whereas the left middle/superior frontal gyri decrease with age, those evidence suggest that the ability of response inhibition increasing with age (Tamm et al., 2002). Larsen et al. (2021) compared the electrophysiological performance of normal-weight and obese individuals (18–40 years). They found that individuals with higher percentages of body fat had larger amplitude on FC_1_ and FT_7_. Indicating that the heavier the body weight, the slower the individual’s response to the stimulus. Studies of sedentary time in overweight and obese individuals found that these people with longer sedentary time showed elevated P3b amplitudes on CPz in the Go/Nogo task. This means those overweight and obese individuals use more attentional resources and have reduced response inhibition in the face of stimuli (Pindus et al., 2021). In addition, other studies of error-related brain activation have found that the anterior and posterior cingulate gyrus, precuneus, and left/right anterior insula cortices are activated during error-only processing (Menon et al., 2001). All of the above studies demonstrate that the frontal and parietal lobes act an important role in the formation and maturation of response inhibition capacity.

In the Stop-Signal task, stop-signal reaction time (SSRT) is the main index used to evaluate inhibition, with longer SSRT indicating poorer response inhibition (Fang et al., 2013). When using the stop-signal compared the response inhibition of obese children in different reward conditions (toy condition and food condition), it was found that their inhibition was worse in the food condition, indicating a deficit in the inhibition of food in obese children (Nederkoorn et al., 2012). However, studies have also reported no differences in SSRT between obese and slim populations (Mühlberg et al., 2016). Additionally, food, body size, and weight have relatively large effects on cognitive function (Kemps et al., 2005), so maintaining a good weight is important for cognitive development. In a review of previous literature, it was found that only six of the 16 studies reported that obese subjects exhibited reduced response inhibition compared to the normal group, and these differences in outcomes appear to be related to the different methods used by the researchers (Renee et al., 2013).

Most of the current national response inhibition researches has been conducted using questionnaires and behavioral measures, and there is a lack of evidence from ERP. In addition, most of such studies have focused on childhood obesity, but response inhibition in children develops with age, and how mature response inhibition relates to obesity in early adulthood remains to be further explored.

Therefore, the Go/Nogo paradigm was chosen to collect EEG data in this study to primarily explore the differences in ERP components between overweight/obese and normal-weight subjects and the deficits in response inhibition ability of obese individuals on EEG results. Behavioral data from the SST task were also used to complement the exploration of the response stopping ability of inhibition.

## Subjects and Methods Headings

### Subjects

There were 58 subjects, all from higher education institutions in Changsha, with age distribution ranging from 18 to 29 years old (control group: 21.43 ± 2.19, overweight–obese group: 21.78 ± 2.72) and BMI values distributed from 18.62 to 40.09 (control group:20.33 ± 1.64, overweight–obese group:27.76 ± 4.07). The subjects were grouped according to the body mass index (BMI) recommended by the 2001 Chinese Working Group on Obesity, with 30 in the control group (12 men and 18 women) and 28 in the overweight–obese group (10 men and 18 women), of whom 16 were overweight and 12 were obese. All subjects were required to be in good health, without chronic diseases (e.g., diabetes and hypertension), without a history of eating disorders and mental illness, excluding overweight or obese due to other diseases, excluding subjects who were dieting to lose weight, vegetarians or with other dietary contraindications (e.g., no pork). All subjects were right-handed, had normal vision or corrected vision, and volunteered to participate in the study.

### Research Method

#### Experimental Materials

Self-Control Scale (SCS): A short-form version of the Self-Control Scale revised by Tan Shuhua consists of 19 items on a five-point scale (1 completely disagree, 5 completely agree). Including 15 reverse scoring items. The higher scores indicate the subject’s greater self-control. This scale has shown good reliability in previous studies with an internal consistency coefficient of 0.862 and a retest reliability of 0.85 (Tan and Guo, 2008).

Barratt Impulsiveness Scale (BIS-11): The BIS-11 revised by Yang Huaiqin consists of 30 items on a four-point scale (1 = almost never/rarely, 2 = occasionally, 3 = often, 4 = almost always/always). It includes 11 reverse scoring items and a total score between 30 and 120, with higher scores representing greater impulsivity. Previous studies reported internal consistency coefficients of 0.77 to 0.89 for this questionnaire, with retest reliability ranging from 0.68 to 0.89 (Yang et al., 2007; Li et al., 2011).

Go/Nogo task: The stimulus material was 20 pictures of food downloaded from the Chinese picture system and the Internet. The pictures were selected by 40 university students after a pre-experiment using a 7-point scale ranking from 28 food pictures. “1” means the lowest calorie, and “7” indicated the highest calorie. The top 10 scores in food pictures are sorted each as highest and lowest. The food pictures were of uniform size of 6.0 cm × 6.0 cm.

SST task: The stimulus material was eight food pictures. The pictures were the standard pictures of 40 college students after scoring the food according to seven levels of calories, and four pictures of each of the highest and lowest scoring food were selected in order of high and low as the materials for the SST task.

#### Experimental Procedure and Design

##### Go/Nogo Tasks

The Go/Nogo task used a 2 (task type: Go task, Nogo task) × 2 (group: control, overweight–obese group) × 3 (electrode sites: N2: Fz, FCz, Cz; P3: Cz, CPz, Pz) repeated measures variance design. The dependent variables were Go/Nogo task error rate, latency, and wave amplitude of N2/P3. Programming was performed using E-prime 2.0, the experimental stimulus was a food picture, and the task required a keypress response. All subjects completed questionnaires and demographic data before the experiment, and then performed the Go/Nogo task, at the same time, EEG signals were collected.

**Figure 1:**
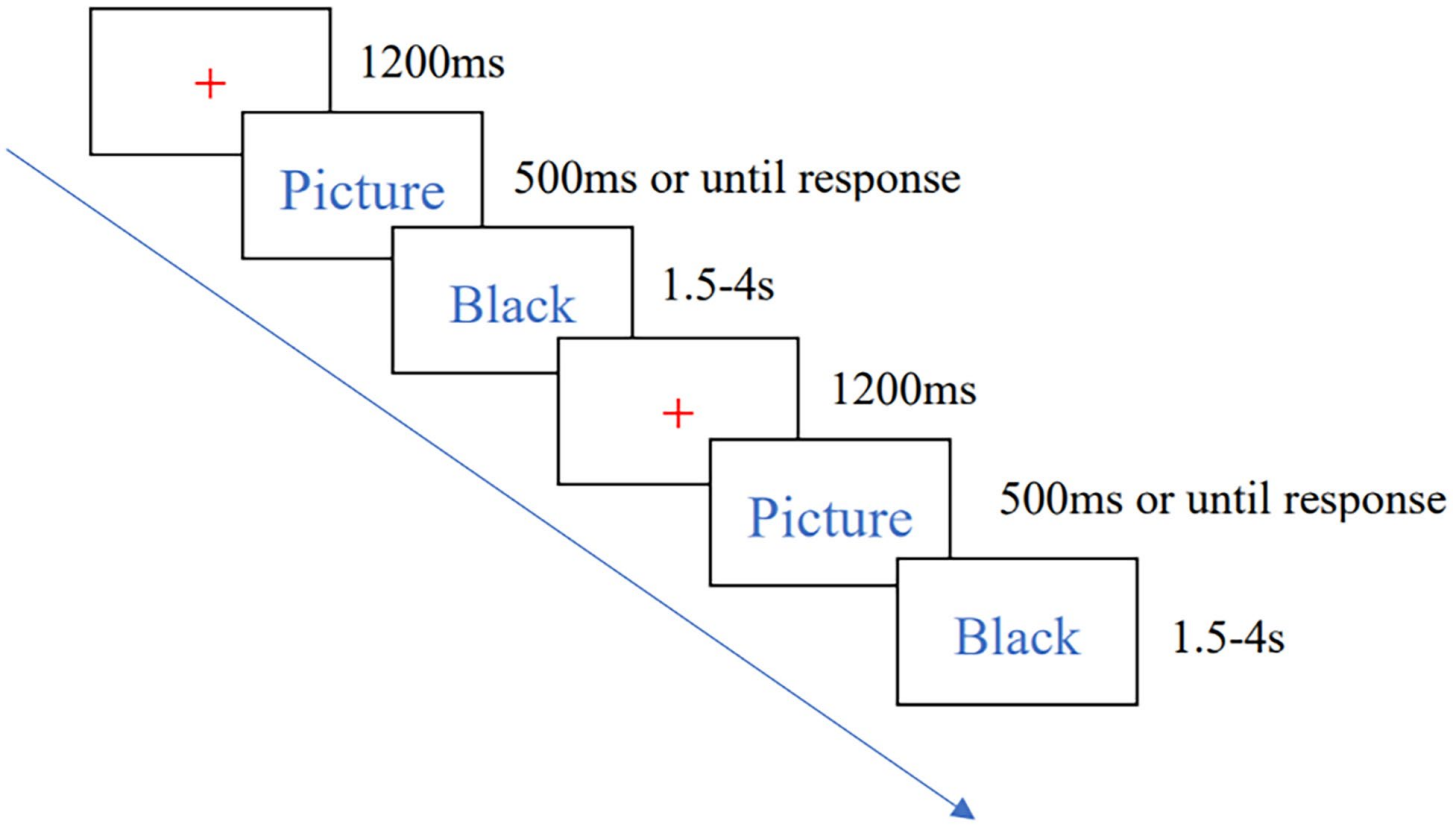
Schematic diagram of Go/Nogo task flow.

The Go/Nogo task consisted of 450 trials and took about 20 min to complete. Subjects were asked to respond by pressing the “J” button when they saw a Go stimulus (low-calorie food pictures, such as tomatoes) and not to respond when they saw a Nogo stimulus (high-calorie food pictures, such as pizza). All stimuli were presented pseudo-randomly. After the task started, a red “+” point appeared in the display and lasted 1,200 ms to remind subjects of the start, followed by the food pictures which presented for 500 ms or until response. And then a black screen lasting 1.5–4 s (average:2.5 s). The flow of a single trial is shown in Figure 1. The formal experiment was practiced before the formal experiment, and the formal experiment started when the correct rate of the subjects reached 80% in the practice phase.

##### SST Tasks

The SST task used a 2 (stimulus type: high-calorie picture, low-calorie picture) × 2 (group: control group, overweight–obese group) repeated measures variance design. The dependent variable was SSRT.

**Figure 2:**
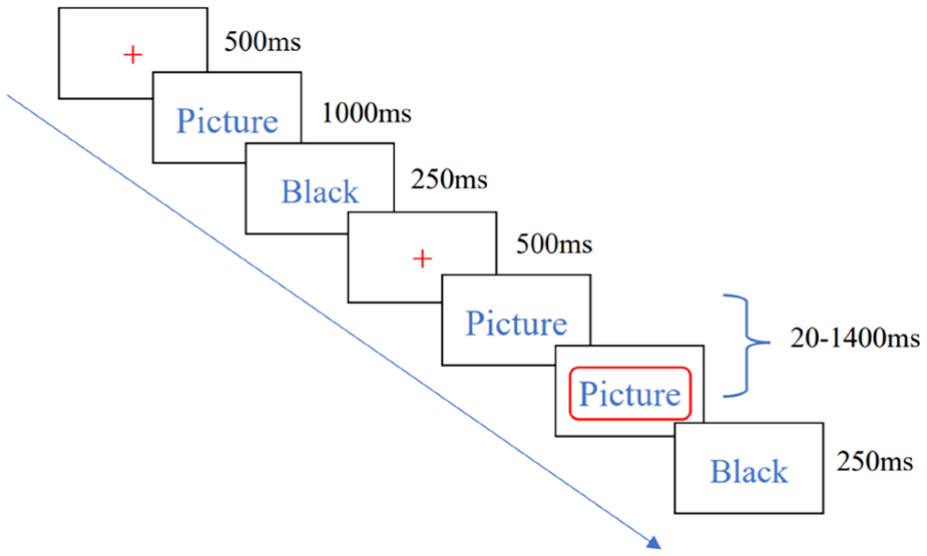
Schematic diagram of SST task flow.

The SST task consisted of 8 blocks, and each block included 40 trials. The entire task lasted approximately 24 min. The experimenter used a 19-inch monitor with 1,024 × 768 pixels to experiment. The whole process includes the Go and Stop two parts. In the Go part, the center of the screen presents red “+” point of attention for 500 ms, then a picture of food lasting 1,000 ms appears on the left or right side of the screen. When the picture was on the left side of the screen, the subject pressed the “F” key with the left hand, and when the picture appeared on the right side of the screen, the subject pressed the “J” key with the right hand, and a black screen will be lasting 250 ms. In the Stop part, the food picture stimulus appeared first on the left or right side of the screen, and then a red box with a stop-signal appeared around the picture, and the subject did not respond to the red box. The interval time between the initial picture and the stop signal was 250 ms. Using the tracking design method, after the subject successfully suppressed the button in the stop task, the next SSD would be increased by 50 ms to increase the difficulty of the subject’s successful suppression, and otherwise reduced by 50 ms to decrease the difficulty of suppression. The high-calorie food pictures and low-calorie food pictures appear the same number of times and are pseudo-randomly presented. The flow of a single trial is shown in Figure 2. The practice was conducted before the formal experiment, and the formal experiment was started when the correct rate of subjects in the practice phase reached 80%.

### Data Recording and Analysis

Using a Neuroscan (Neurosoft, Inc. Sterling, United States) polyconductance EEG evoked potential workstation recorded EEG waves simultaneously during the Go/Nogo task. The conduction method used was the International 10–20 system, using Ag/AgCl electrodes with 64 conductive pole caps. The left ear unilateral mastoid (M1) was used as the reference electrode to record horizontal and vertical EEG through four active electrodes, which were used for the removal of oculogram artifacts for subsequent offline analysis of EEG signals. The EEG was acquired by AC with a filtered bandpass of 0.05 to 100 Hz and a sampling frequency of 1,000 Hz/conductance, amplified by an amplifier and recorded as continuous EEG.

Using SCAN 4.7 analyzed offline data of EEG data. The data of 25 subjects with too much resistance and artifacts, detached external electrodes, and less than 30 EEG superimpositions were excluded. Also, oculomotor artifacts and muscle movement artifacts formed by involuntary blinking and swallowing of saliva were excluded. The EEG segments were taken at 200 ms before and 1,000 ms after the stimulus. The EEG at 200 ms before the stimulus is the corrected baseline. The artifact removal standard was set to ±100 μV, and digital filtering was performed using a phase-shift-free bandpass of 30 Hz (24 dB/oct). The total average was superimposed for each experimental condition and group. The time windows of the two components were determined according to the total averaging plot and previous literature (Zhao and Zheng, 2001; Dang et al., 2004; Ji and Zhang, 2008), with the N2 and P3 component appearing from 180–350 ms to 300–550 ms separately. Three electrode points (Fz, FCz, and Cz) were selected according to the head position where the N2 component appeared, and three electrode points (Cz, CPz, and Pz) were selected for the P3 component (Bokura et al., 2001; Liddle et al., 2001; Zhang et al., 2012). Using wave detection in SCAN 4.7 software, the latencies and wave amplitudes of the N2 and P3 components for all subjects in different conditions were exported. These data were analyzed by repeated measures ANOVA and simple effects performed.

Behavioral experimental task data and questionnaire data were collated and exported to SPSS17.0. Excluded the unqualified data and calculated the correct Go sub-trial mean response time, Go omission errors, commission errors of the Nogo task, SSRT, SCS scores, and BIS scores. The analysis methods were repeated measures ANOVA, chi-square test, t-test, Pearson’s correlation analysis. The correction method in the repeated measures ANOVA was Greenhouse–Geisser, and *p* < 0.05 was considered as a statistically significant difference.

## Results

### Behavioral Data Results

#### Go/Nogo Task Results

Behavioral data and questionnaire data from the two groups of subjects were compared using independent samples *t*-tests. The comparisons of SCS, BIS-11, Go omission errors, Nogo commission errors, and Go task response time in the Go/Nogo task as shown in Table 1 below. There were no significant differences between the two groups at SCS, BIS-11, Go task response time (*p* > 0.05), and Go task omission errors (*p* = 0.663). However, the commission errors of the Nogo task were significantly higher in the overweight–obese group than in the normal control group (*p* = 0.002). As shown in Table 1.

**Table 1:** Comparison of results between control and overweight–obese groups on each scale score and Go/Nogo task.

Variables	Control group (*n* = 30)	Overweight–obese group (*n* = 28)	*t*	*p*
SCS	59.47 ± 11.36	61.07 ± 8.64	−0.602	0.549
BIS-11	64.97 ± 6.59	67.96 ± 11.45	−1.232	0.223
Response time (GO)	424.41 ± 17.58	414.77 ± 21.08	0.202	0.063
Omission errors (GO)	0.198 ± 0.10	0.18 ± 0.16	0.438	0.663
Commission errors (Nogo)	0.13 ± 0.05	0.19 ± 0.09	−3.323	0.002

To further explore the relationship of behavioral data, 58 subjects were analyzed by Pearson correlation analysis, which revealed that Go task omission errors were significantly negatively correlated with Nogo task commission errors (*p*_Go task_ = 0.003). Go task response time was positively correlated with omission errors while negatively correlated with Nogo commission errors; BMI value was significantly positively correlated with Nogo commission errors (*p* = 0.001). As shown in Table 2.

**Table 2:** Correlation analysis between scale scores and Go/Nogo tasks.

	Omission errors (GO)	Commission errors (Nogo)	Response time (GO)	BIS-11	SCS	BMI
Omission errors (GO)	1					
Commission errors (Nogo)	−0.379^**^	1				
Response time (GO)	0.754^**^	−0.593^**^	1			
BIS-11	0.143	0.158	0.042	1		
SCS	0.000	−0.309^*^	0.093	−0.135	1	
BMI	0.060	0.409^**^	−0.139	0.191	0.128	1

^*^Indicates significant correlation at the 0.05 level (bilateral); ^**^Indicates significant correlation at the 0.01 level (bilateral).

#### SST Task Results

After excluding two subjects with too low correct rate (<30%), the task left 56 subjects, 29 in the control group (11 males and 18 females) and 27 in the overweight–obese group (10 males and 17 females). A repeated measures ANOVA on stop-signal response time (SSRT) revealed a significant interaction between group and stimulus type, (F_1,54_ = 7.675, *p* = 0.008). Further simple effects analysis revealed that SSRT for the high-calorie food picture stimulus was significantly greater than low-calorie stimulus in both of control and overweight–obese groups (*p* < 0.001). However, there was no significant difference between the two groups for the low-calorie food picture stimulus (*p* = 0.076). As for the high-calorie food picture stimulus, the SSRT was significantly greater in the overweight–obese group than in the control group (*p* = 0.006).

Similarly, repeated measures analysis of response time revealed that the interaction between group and stimulus type was not significant (*F*_1,54_ = 2.548, *p* = 0.116), but the main effect of the stimulus was significant (*F*_1,54_ = 107.896, *p* < 0.001). After fixing group category, the response time of low-calorie stimulus was smaller than high-calorie stimulus in both groups (p < 0.001). And after fixing the stimulus category, the response time of the overweight–obese group was greater than the control group under both stimuli (*p*_low calorie_ = 0.025, *p*_high calorie_ = 0.017). As shown in Table 3.

**Table 3:** Comparison between the control group and overweight–obese group at the time of stopping signal response and at the time of response.

	Control group (*n* = 29)		Overweight–obese group (*n* = 27)	
	Low-calorie	High-calorie	Low-calorie	High-calorie
SSRT	290.49 ± 37.17	302.77 ± 38.77	308.12 ± 35.61	332.16 ± 38.10
Go RT	612.93 ± 114.88	629.16 ± 116.81	674.98 ± 83.02	697.09 ± 86.03

### ERP Results

**Figure 3:**
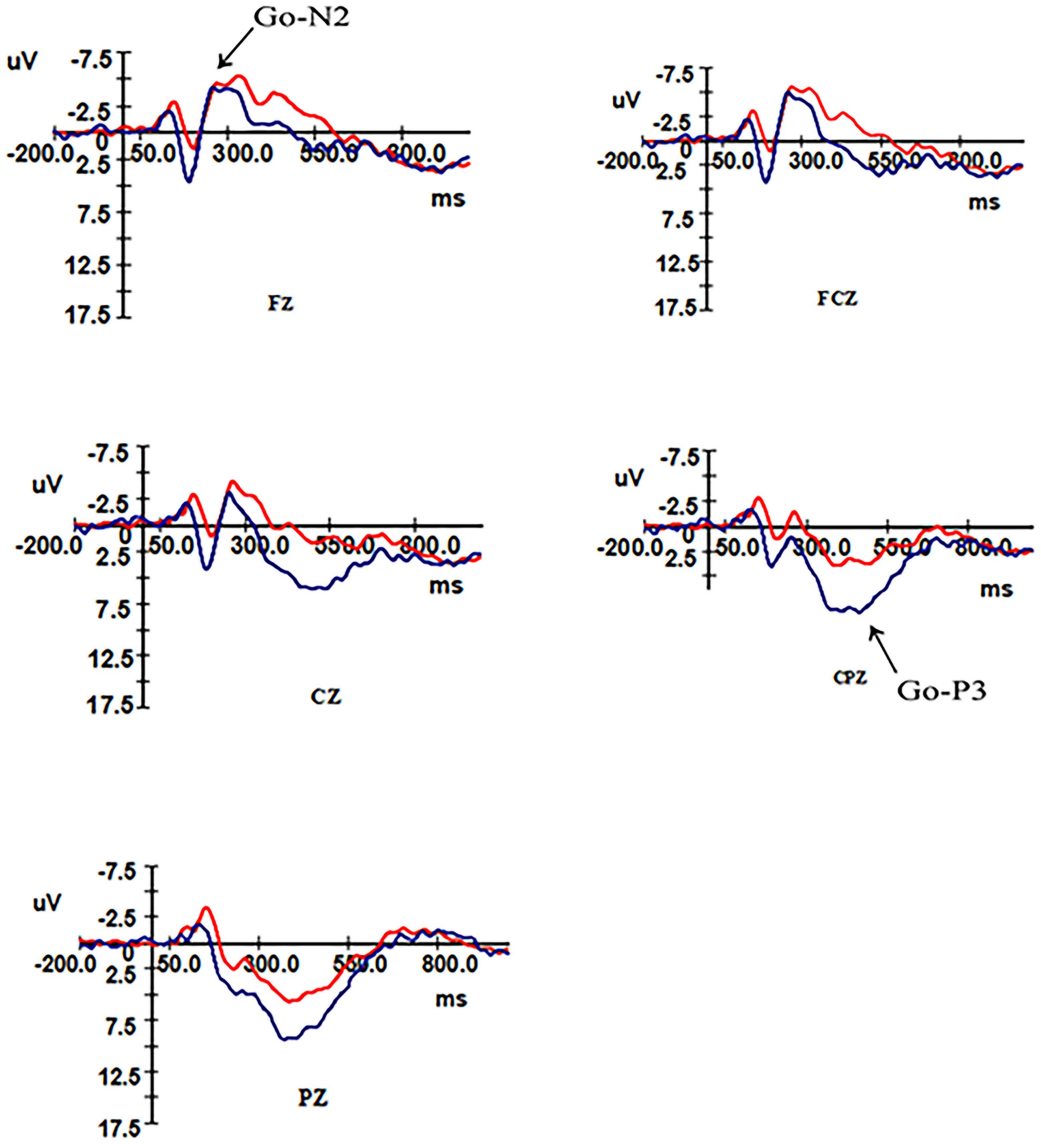
Total mean EEG components in control and overweight obese groups under Go stimulation.

**Figure 4:**
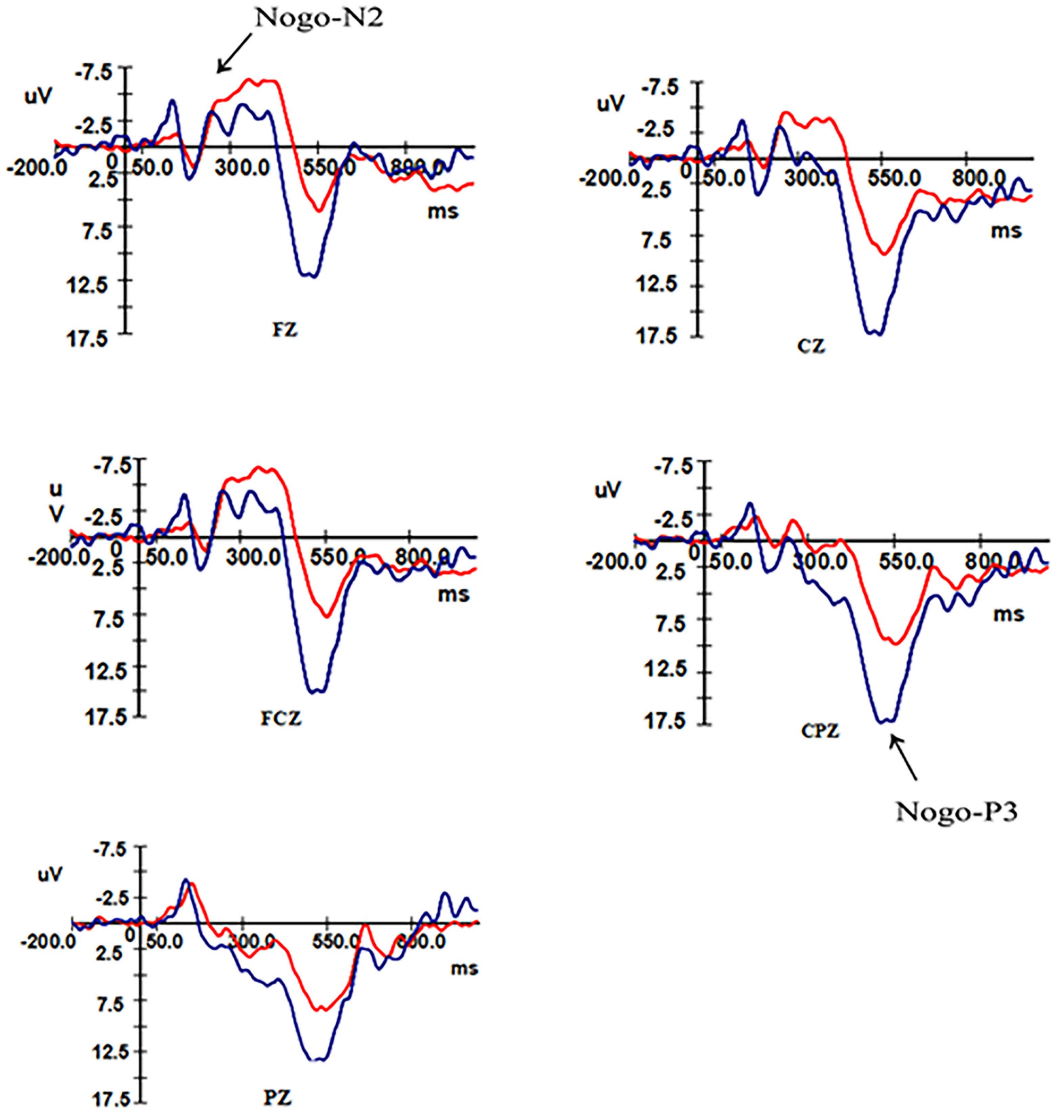
Total mean EEG components in the control and overweight obese groups under Nogo stimulation.

After removing the ineligible subjects 16 (six males and 10 females) remained in the control group and 17 (eight males and nine females) in the overweight–obese group. Analysis of the N2 and P3 components triggered by the high-calorie and low-calorie food pictures revealed that both the control group and the overweight–obese group successfully induced the two components during the two-time windows of 180 ms ~ 350 ms and 300 ms ~ 550 ms after stimulation, and the total average graphs are shown in Figures 3, 4.

#### N2 Component Results

ANOVA was performed on the wave amplitude of the N2 component: the main effect of task type (*F*_1,31_ = 4.958, *p* = 0.033) and electrode point (*F*_2,30_ = 47.31, *p* < 0.001) were significant. Fixed group and task conditions revealed that N2 wave amplitude was greater under the Nogo task than the Go task. The difference in wave amplitude between Fz and FCz electrode sites was not significant (*p* = 0.337), but the above electrode sites’ wave amplitude was significantly greater than Cz electrode sites (*p* < 0.01).

ANOVA was performed on the latency of the N2 component: the main effect of electrode site was significant (*F*_2,30_ = 14.882, *p* < 0.01), no significant difference was found for the fixed group (*p* = 0.979) in latency between the two groups, and no significant difference was found for fixed electrode sites Fz and FCz electrode sites (*p* = 0.214) in latency, but they were both significantly greater than the latency for Cz electrode sites (*p* < 0.01). As shown in Table 4.

**Table 4:** Comparison of the results of N2 component wave amplitude and latency in the control and overweight–obese groups.

	Electrode sites	Control group (*n* = 16)		Overweight–obese group (*n* = 17)	
		Go task	Nogo task	Go task	Nogo task
Amplitude (uv)	Fz	−7.05 ± 5.84	−8.49 ± 7.35	−7.06 ± 4.41	−6.84 ± 4.40
	FCz	−7.11 ± 5.34	−8.73 ± 7.09	−7.60 ± 3.69	−7.98 ± 3.55
	Cz	−4.48 ± 4.10	−5.87 ± 6.19	−5.60 ± 2.99	−6.37 ± 3.22
Latency (ms)	Fz	292.00 ± 34.88	297.31 ± 44.80	301.35 ± 42.55	305.18 ± 37.96
	FCz	286.63 ± 33.19	297.63 ± 42.99	298.18 ± 40.49	304.47 ± 39.28
	Cz	268.88 ± 36.11	271.25 ± 43.99	279.35 ± 52.23	283.65 ± 53.57

#### P3 Component Results

An ANOVA was performed on the wave amplitudes of the P3 component: a simple effects analysis was performed, fixing group for the three electrode sites in different tasks, with a significant P3 effect for the control group and overweight–obese group where the wave amplitude of the Nogo task was greater than that of the Go task for all three electrode sites. When the two groups were compared at fixed task type, the control group wave amplitude was more positive than the overweight–obese group in both Go task type and Nogo task type (*p*_Go task_ = 0.014, *p*_Nogo task_ = 0.002). When different electrode points of both groups were analyzed at fixed task types, the wave amplitudes of both Cz and CPz electrode points were significantly greater in the Go task type than in the overweight–obese group (*p*_Cz_ = 0.007, *p*_CPz_ = 0.013), and the wave amplitudes of Pz electrode points were also significantly greater in the control group than in the overweight–obese group (*p*_Pz_ = 0.059); in the Nogo task type, all three electrode point wave amplitudes were significantly larger in the control group than in the overweight–obese group (*p* < 0.05).

ANOVA was performed on the latency of the P3 component: the main effect of task type was significant (*F*_1,31_ = 10.837, *p* = 0.002), and the latency under the Go task was smaller than the Nogo task. As shown in Table 5.

**Table 5:** Comparison of the results of P3 component wave amplitude and latency in the control and overweight–obese groups.

	Electrode sites	Control group (*n* = 16)		Overweight–obese group (*n* = 17)	
		Go task	Nogo task	Go task	Nogo task
Amplitude (uv)	Cz	8.40 ± 5.84	19.16 ± 8.33	3.67 ± 4.97	10.80 ± 5.87
	CPz	10.92 ± 5.78	19.33 ± 7.77	6.09 ± 4.77	11.58 ± 4.19
	Pz	11.35 ± 7.06	15.71 ± 7.90	7.34 ± 4.50	10.59 ± 3.49
Latency (ms)	Cz	469.31 ± 74.36	514.93 ± 46.20	458.12 ± 94.87	506.53 ± 79.29	
	CPz	494.50 ± 64.88	529.50 ± 23.68	466.18 ± 89.35	517.47 ± 61.00	
	Pz	495.56 ± 52.17	530.50 ± 24.14	459.53 ± 88.42	516.41 ± 60.36

## Discussion

### Analysis of Behavioral Results of Go/Nogo Task Results

In this study, there was no significant difference between the response time of the two groups of subjects, indicating that the two groups had the same response speed. The commission error in the Nogo task of high-calorie food pictures was significantly greater in the overweight–obese group than in the control group. The commission errors in the Nogo stimulus are the main evaluation index of the experiment, and a higher rate of commission errors means a weaker inhibition ability. What’s more, BMI was positively correlated with Nogo commission error, with heavier subjects having higher commission errors. This supports the hypothesis of this study that overweight and obese individuals have a weaker ability to inhibit pictures of high-calorie foods.

The analysis of the questionnaire results revealed no significant differences between the two groups in the SCS and BIS-11 scores. However, there are differences between the two groups of subjects in Go/Nogo results. This means that behavioral data can reduce some extent error of subjects in self-reporting due to social probability and subjectivity. Thus, behavioral measurements have higher objectivity.

Go trial omission errors and Nogo trial commission errors were negatively correlated. The more omissions on Go trials and the fewer commissions on Nogo trials indicated that subjects tended to adopt a “conservative” strategy in conducting the experiment, with longer response times and more non-responses. In contrast, the lower number of omission errors of Go trials and the higher number of commission errors of Nogo trials, the more subjects tended to adopt an “aggressive” strategy, with shorter response times and more total keystroke responses. This difference in strategy choice may be the result of a trade-off between the subject’s past behavioral habits and speed accuracy and is not strongly related to body weight.

### Analysis of Behavioral Results of Stop-Signal Task

The automatic tracking design chosen in this study can adjust the SSD duration for the next trial timely, which can reflect the subjects’ true inhibition ability better and is more adaptable to individual variability. In addition, the SSRT calculation in this method is easier than directly setting several fixed SSD durations. In this study, using repeated measures ANOVA, it was found that SSRT with high-calorie food picture stimuli was significantly greater than SSRT with low-calorie food picture stimuli for both groups of subjects, indicating that both normal and overweight–obese groups had lower inhibitory ability to high-calorie food picture stimuli than low-calorie food pictures. And all subjects preferred high-calorie foods, which is also consistent with the evolutionary perspective. However, fixing the stimulus type to compare the two groups of subjects, the difference between the two groups in the low-calorie food picture condition was not significant, indicating that the late inhibitory ability to low-calorie food was similar for both groups. In the high-calorie food picture condition, the SSRT was significantly longer in the overweight–obese group than in the control group, indicating that the overweight–obese group had a lower inhibitory capacity to high-calorie food.

The results of the analysis of the response time to different heat picture stimuli revealed that the low-calorie stimulus–response time was shorter than the high-calorie stimulus in both groups of subjects. Considering our experimental sequence arrangement, all subjects completed the Go/Nogo experiment first and then performed the SST experiment. In the Go/Nogo experiment, subjects were required to respond to high-frequency low-calorie stimuli and not to low-frequency high-calorie stimuli, this rule was internalized in the SST task later. That means even the occurrence probability of high and low-caloric stimuli in the SST task was the same, subjects still have longer response times to high-caloric stimuli than low-caloric stimuli in both groups in this experiment.

After fixing the stimulus categories, it was found that the reaction time of the overweight and obese subjects was longer than the control subjects in both stimulus conditions. This part of task did not require subjects to identify and process whether the picture was a high-calorie food or a low-calorie food, but only to process the position of the picture. The differences between the two groups showed shortcomings in the subjects’ processing speed.

### ERP Component Analysis

#### N2 Component Analysis

The results of this study found that the Go-N2 and Nogo-N2 wave amplitudes differed between conditions, with the Go-N2 wave amplitude significantly smaller than the Nogo-N2 wave amplitude in the control and overweight–obese groups, and the N2 wave amplitude was higher under the difficult task with a significant N2 effect emerged, consistent with the results of previous studies (Reyes et al., 2015). The interaction effect between task type and group was not significant, and the difference in N2 wave amplitude triggered by either Go or Nogo stimuli between the control and overweight–obese groups was not significant, indicating that there was no difference between the two groups on early conflict monitoring. All subjects in this study were adults, and as people grow up, the brain gradually develops and matures, the conflict monitoring ability is gradually improved. So, the difference in ability between the two groups in adulthood is not significant, and compared with children, adult individuals can more easily detect external stimulus conflict and identify the stimulus type. In the experiment, both control and overweigh–obese group subjects were able to recognize and process the stimuli when they saw pictures of high and low-calorie foods. When the three electrode sites were analyzed, it was found that the wave amplitudes at both Fz and FCz were significantly larger than those at Cz, indicating that the N2 component was mainly present in the precentral frontal area.

Analysis of the latency of the N2 component revealed no significant differences between the latencies of the different conditions. And there was also no significant difference between the latencies of the two groups under the same conditions. The above can indicate that the early conflict monitoring mechanism was normal in the overweight–obese group in this study.

#### P3 Component Analysis

This study found that the main effect of different task types was significant, and the P3 wave amplitude induced by Nogo stimulation was significantly larger than that induced by Go stimulation, and a significant P3 effect emerged. After Go stimulation, there were significant group differences in P3 wave amplitude at both electrode sites and a borderline significant difference at the other electrode site, and the Go-P3 wave amplitude in the control group was higher than the Go-P3 wave amplitude in the overweight–obese group. After the Nogo stimulus appearing, the P3 amplitude in the control group was significantly larger than the P3 amplitude in the overweight–obese group, similar to previous studies (Tascilar et al., 2011; Kamijo and Masaki, 2016). The greater the P3 amplitude, the stronger the inhibition, so that normal individuals have a stronger inhibition than overweight–obese individuals in response to high and low-calorie food stimuli. It is suggested that overweight/obese individuals have some deficit in response behavior inhibition in the face of food-related stimuli.

Latency analysis of the P3 component revealed significant main effects under different conditions, with Go-P3 latency times smaller than Nogo-P3. However, P3 latency was not significant for the main effect of group, the main effect of electrode site, or the interaction effect of group, electrode site, and task type. Therefore, there was no difference in P3 latency between the two groups of subjects. That is, the overweight–obese subjects did not differ significantly from the control group in processing speed. A possible explanation for this is that the low-calorie food pictures appeared as Go stimuli with a frequency of 75% in this study, which is a high-frequency stimulus. The frequent occurrence of low-calorie food pictures made the task less difficult, the practice effect was more obvious, and the subjects processed the low-calorie pictures more easily and skillfully. So all subjects were more responsive to low-calorie pictures relative to the high-calorie pictures. Meanwhile, the behavioral task in this experiment was not too difficult for both groups of subjects. Thus, although the P3 component was both excited, it is difficult to detect the difference.

### Summary

In summary, there were no significant differences between the control and overweight–obese groups in the wave amplitude and latency of the N2 component. That indicating that the overweight–obese subjects in this experiment had little impairment in early inhibitory processing and conflict monitoring. Although there was no significant difference between the two on conflict monitoring, the overweight–obese subjects in the experiment performed poorly on behavioral control. This was verified by the significant reduction in P3 component amplitude in the overweight–obese group: the overweight–obese subjects had reduced inhibitory ability in behavioral control. Overweight–obese individuals showed poor behavioral control mainly performed as even when they did not need to eat, overweight–obese subjects still had difficulty in inhibiting their eating behaviors in the face of food stimuli. And this situation was more obvious in the face of high-calorie foods. As a result, the overweight–obese subjects consumed more energy, resulting in higher body weight. The analysis of SST reaction time showed that although both normal subjects and overweight–obese subjects had lower inhibitory ability to high-calorie foods, overweight–obese subjects had longer reaction time and slower reaction speed when faced with high-calorie food, so their reaction inhibition ability was lower and their processing speed was somewhat impaired. In general, the behavioral data results are more consistent with the EEG results. In both results, it can be concluded that the overweight–obese group has lower response inhibition than the control group.

Behavioral measures are easy to manipulate and have relatively objective results to certain stimuli. And EEG studies can clarify different stages of response. Each of the two measurement modalities has its focus. However, there are drawbacks to these types of research. Behavioral experiments may have subjects who do not understand the requirements of the experiment. And if the experiment is too long, it may lead to subject fatigue, reduced attention and willpower, etc. The EEG study needs to be balanced between quality and quantity, and the preparation of the experiment is tedious.

Also, two main conclusions were drawn from this study.

1. In terms of behavioral outcomes, overweight/obese subjects had deficits in dominant response inhibition and response cessation compared to normal-weight subjects. This is in line with previous studies where subjects with weaker behavioral inhibition ate more (Jiang, 2010) and obese subjects showed weaker inhibition in food-only-related settings (Bartholdy et al., 2016).
2. In terms of EEG results, overweight/obese subjects showed no difference in processing speed and level of conflict monitoring for early inhibitory processing compared to normal-weight subjects, but deficits in behavioral control for late inhibitory processing.

Both of these findings suggest that there are indeed some problems with response inhibition in overweight/obese subjects, contributing to our understanding of the causes of simple obesity while adding to the theoretical basis for response inhibition in the obese population. In addition, the study findings provide empirical information for intervention and treatment of overweight obesity, suggesting that the general public should pay attention to the functional impairment produced by overweight obesity in individuals and strengthen the importance of obesity in society. One researcher found that the inhibitory control of the overweight–obese group was improved by a two-week intervention and training, the wave of P3 became larger, and the intake of healthy foods increased while the intake of unhealthy foods became less in these subjects who participated in the intervention training in real life (Blackburne et al., 2016). This suggests that we can use the amplitude of P3 as an indicator of post-intervention effects. The intervention improves the individual’s response inhibition and develops healthy food choice habits, which in turn reduces the individual’s body weight.

Although our study investigated the response inhibition of overweight/obese people, there are many shortcomings: (1) this study chose college students as the study subjects, at the same time, in order to try to ensure the quality of EEG data, many unqualified subjects’ data were excluded. Thus, the sample size was small, the sample source was relatively fixed, and the representativeness was not enough; (2) In this study, BMI was used as the basis for judging overweight or obesity, but it would be more accurate if other indicators, such as body fat percentage, waist circumference, and waist–hip ratio, were added together to judge the screening of overweight and obese subjects; (3) In this study, although many measures were taken to minimize scalp resistance during the preparation of EEG data collection, there were still some subjects with unsatisfactory resistance reduction effect, which also affected the accuracy of the data.

## Data Availability Statement

The raw data supporting the conclusions of this article will be made available by the authors, without undue reservation.

## Ethics Statement

The studies involving human participants were reviewed and approved by Institutional Review Board of The Second Xiangya Hospital of Central South University. The patients/participants provided their written informed consent to participate in this study.

## Author Contributions

T-SC designed the whole study and followed up all the procedures. Z-NL contributed to the data collection and analysis, manuscript writing of the article. J-YJ contributed to the data collection, manuscript writing, and photo production process of the article. D-LZ made certain contributions in data collection and data analysis. Z-NL, T-SC, J-YJ, and D-LZ were involved in the study conduction and contributed to the revision and deletion of the article. All authors contributed to the article and approved the submitted version.

## Funding

This work was supported by the Natural Science Foundation of Hunan Province: ERP Study on Brain Mechanism of Emotional Eating in Obese Adolescents (Number: 420500014), Hunan Provincial Education Science “Twelfth Five-Year Plan” Project: Psychological Intervention Research on Transtheoretical Model of Emotional Eating in Obese Adolescents (Number: XJK014BXL007), and Hunan Provincial Philosophy and Social Science Foundation Project: Psychological Intervention of Emotional Eating in Obese Adolescents (Number: 13YBA340).

## Conflict of Interest

The authors declare that the research was conducted in the absence of any commercial or financial relationships that could be construed as a potential conflict of interest.

## Publisher’s Note

All claims expressed in this article are solely those of the authors and do not necessarily represent those of their affiliated organizations, or those of the publisher, the editors and the reviewers. Any product that may be evaluated in this article, or claim that may be made by its manufacturer, is not guaranteed or endorsed by the publisher.

## References

[ref1] Aron A. R. (2011). From reactive to proactive and selective control: developing a richer model for stopping inappropriate responses. Biol. Psychiatry 69, e55–e68. doi: 10.1016/j.biopsych.2010.07.024, PMID: 20932513 PMC3039712

[ref2] Bari A. Robbins T. W. (2013). Inhibition and impulsivity: behavioral and neural basis of response control. Prog. Neurobiol. 108, 44–79. doi: 10.1016/j.pneurobio.2013.06.005, PMID: 23856628

[ref3] Bartholdy S. Dalton B. O’Daly O. G. Campbell I. C. Schmidt U. (2016). A systematic review of the relationship between eating, weight and inhibitory control using the stop signal task. Neurosci. Biobehav. Rev. 64, 35–62. doi: 10.1016/j.neubiorev.2016.02.010, PMID: 26900651

[ref4] Batterink L. Yokum S. Stice E. (2010). Body mass correlates inversely with inhibitory control in response to food among adolescent girls: an fMRI study. NeuroImage 52, 1696–1703. doi: 10.1016/j.neuroimage.2010.05.059, PMID: 20510377 PMC2910204

[ref5] Blackburne T. Rodriguez A. Johnstone S. J. (2016). A serious game to increase healthy food consumption in overweight or obese adults: randomized controlled trial. JMIR Serious Games 4:e10. doi: 10.2196/games.5708, PMID: 27417192 PMC4963607

[ref6] Blume M. Schmidt R. Hilbert A. (2018). Executive functioning in obesity, food addiction, and binge-eating disorder. Nutrients 11:54. doi: 10.3390/nu11010054, PMID: 30597858 PMC6356459

[ref7] Bokura H. Yamaguchi S. Kobayashi S. (2001). Electrophysiological correlates for response inhibition in a Go/Nogo task. Clin. Neurophysiol. 112, 2224–2232. doi: 10.1016/s1388-2457(01)00691-5, PMID: 11738192

[ref8] Calle E. E. Rodriguez C. Walker-Thurmond K. Thun M. J. (2003). Overweight, obesity, and mortality from cancer in a prospectively studied cohort of U.S. adults. N. Engl. J. Med. 348, 1625–1638. doi: 10.1056/NEJMoa021423 12711737

[ref9] Contu L. Hawkes C. A. Bakovic M. (2017). A review of the impact of maternal obesity on the cognitive function and mental health of the offspring. Int. J. Mol. Sci. 18:1093. doi: 10.3390/ijms18051093, PMID: 28534818 PMC5455002

[ref10] Dang Y. J. Wang Y. H. Wang Y. P. (2004). Advances in the study of event-related potentials and clinical applications. Chin. J. Rehabil. Theory Pract. 5, 36–37. doi: 10.3969/j.issn.1006-9771.2004.05.013, PMID: 23020641

[ref11] Deitel M. Greenstein R. J. (2003). Recommendations for reporting weight loss. Obes. Surg. 13, 159–160. doi: 10.1381/096089203764467117 12760387

[ref12] Diamond A. (2013). Executive functions. Annu. Rev. Psychol. 64, 135–168. doi: 10.1146/annurev-psych-113011-143750, PMID: 23020641 PMC4084861

[ref13] Dimoska A. Johnstone S. J. Barry R. J. (2006). The auditory-evoked N2 and P3 components in the stop-signal task: indices of inhibition, response-conflict or error-detection? Brain Cogn. 62, 98–112. doi: 10.1016/j.bandc.2006.03.011 16814442

[ref14] Dougherty D. M. Mathias C. W. Marsh D. M. Jagar A. A. (2005). Laboratory behavioral measures of impulsivity. Behav. Res. Methods 37, 82–90. doi: 10.3758/bf03206401 16097347

[ref15] Enriquez-Geppert S. Konrad C. Pantev C. Huster R. J. (2010). Conflict and inhibition differentially affect the N200/P300 complex in a combined Go/Nogo and stop-signal task. NeuroImage 51, 877–887. doi: 10.1016/j.neuroimage.2010.02.043, PMID: 20188191

[ref16] Falkenstein M. Hoormann J. Hohnsbein J. (1999). ERP components in Go/Nogo tasks and their relation to inhibition. Acta Psychol. 101, 267–291. doi: 10.1016/s0001-6918(99)00008-6, PMID: 10344188

[ref17] Fang J. Zhu Y. Zhao W. Zhang B. Wang X. (2013). Stop signal task and the related models of response inhibiton. Chin. J. Clin. Psychol. 21, 743–746. doi: 10.16128/j.cnki.1005-3611.2013.05.030

[ref18] Flegal K. M. Graubard B. I. Williamson D. F. Gail M. H. (2018). Excess deaths associated with underweight, overweight, and obesity: an evaluation of potential bias. Vital Health Stat. 3 42, 1–21. 30216148

[ref19] Hasselbalch B. J. Knorr U. Kessing L. V. (2011). Cognitive impairment in the remitted state of unipolar depressive disorder: a systematic review. J. Affect. Disord. 134, 20–31. doi: 10.1016/j.jad.2010.11.011, PMID: 21163534

[ref20] Ji X. Zhang J. S. (2008). Application of event-related potential N2 in the study of executive function in children. Chin. J. Child Health Care 16, 554–556. doi: 10.3969/j.issn.1008-6579.2008.05.024

[ref21] Jiang X. X. (2010). The influence of inhibition control and sensitivity to reward on eating behavior. master’s thesis. Chongqing: Southwest University.

[ref22] Kamijo K. Masaki H. (2016). Fitness and ERP indices of cognitive control mode during task preparation in preadolescent children. Front. Hum. Neurosci. 10:441. doi: 10.3389/fnhum.2016.00441, PMID: 27625604 PMC5003924

[ref23] Keita K. (2015). Association between childhood obesity and ERP measures of executive control. J. Phys. Fitness Sports Med. 4, 103–106. doi: 10.7600/jpfsm.4.103

[ref24] Kemps E. Tiggemann M. Marshall K. (2005). Relationship between dieting to lose weight and the functioning of the central executive. Appetite 45, 287–294. doi: 10.1016/j.appet.2005.07.002, PMID: 16126305

[ref25] Larsen B. A. Klinedinst B. S. Grundy J. Willette A. A. (2021). Electrophysiological examination of executive functioning in lean and obese younger adults. Alzheimers Dement. 17:e058743. doi: 10.1002/alz.058743

[ref26] Li X. Y. Philips M. R. Xu D. Zhang Y. L. Yang S. J. Tong Y. S.. (2011). Reliability and validity of an adapted Chinese version of Barratt impulsiveness scale. Chin. Ment. Health J. 25, 610–615. doi: 10.3969/j.issn.1000-6729.2011.08.013

[ref27] Liddle P. F. Kiehl K. A. Smith A. M. (2001). Event-related fMRI study of response inhibition. Hum. Brain Mapp. 12, 100–109. doi: 10.1002/1097-0193(200102)12:2<100::aid-hbm1007>3.0.co;2-6 11169874 PMC6871906

[ref28] Liu Y. J. (2020). Report on the state of nutrition and chronic diseases in China (2020). Food Nutr. China 12:2. doi: 10.19870/j.cnki.11-3716/ts.2020.12.001

[ref29] Luna B. (2009). Developmental changes in cognitive control through adolescence. Adv. Child Dev. Behav. 37, 233–278. doi: 10.1016/s0065-2407(09)03706-9 19673164 PMC2782527

[ref30] Menon V. Adleman N. E. White C. D. Glover G. H. Reiss A. L. (2001). Error-related brain activation during a Go/Nogo response inhibition task. Hum. Brain Mapp. 12, 131–143. doi: 10.1002/1097-0193(200103)12:3<131::aid-hbm1010>3.0.co;2-c, PMID: 11170305 PMC6872006

[ref31] Mora-Gonzalez J. Esteban-Cornejo I. Cadenas-Sanchez C. Migueles J. H. Rodriguez-Ayllon M. Molina-García P.. (2019). Fitness, physical activity, working memory, and neuroelectric activity in children with overweight/obesity. Scand. J. Med. Sci. Sports 29, 1352–1363. doi: 10.1111/sms.13456 31058358

[ref32] Mühlberg C. Mathar D. Villringer A. Horstmann A. Neumann J. (2016). Stopping at the sight of food – how gender and obesity impact on response inhibition. Appetite 107, 663–676. doi: 10.1016/j.appet.2016.08.121, PMID: 27592420

[ref33] Nederkoorn C. Coelho J. S. Guerrieri R. Houben K. Jansen A. (2012). Specificity of the failure to inhibit responses in overweight children. Appetite 59, 409–413. doi: 10.1016/j.appet.2012.05.028, PMID: 22664299

[ref34] Pindus D. M. Edwards C. G. Walk A. M. Reeser G. Burd N. A. Holscher H. D.. (2021). Sedentary time is related to deficits in response inhibition among adults with overweight and obesity: an accelerometry and event-related brain potentials study. Psychophysiology 58:e13843. doi: 10.1111/psyp.13843, PMID: 34021599

[ref35] Quek Y.-H. Tam W. W. S. Zhang M. W. B. Ho R. C. M. (2017). Exploring the association between childhood and adolescent obesity and depression: a meta-analysis. Obes. Rev. 18, 742–754. doi: 10.1111/obr.12535, PMID: 28401646

[ref36] Renee C. Joshua H. Melissa H. (2013). Attentional bias and response inhibition in obese individuals: a systematic review. Obes. Res. Clin. Pract. 7:e95. doi: 10.1016/j.orcp.2013.12.672, PMID: 24331771

[ref37] Reyes S. Peirano P. Peigneux P. Lozoff B. Algarin C. (2015). Inhibitory control in otherwise healthy overweight 10-year-old children. Int. J. Obes. 39, 1230–1235. doi: 10.1038/ijo.2015.49, PMID: 25869603 PMC4526395

[ref38] Skoranski A. M. Most S. B. Lutz-Stehl M. Hoffman J. E. Hassink S. G. Simons R. F. (2013). Response monitoring and cognitive control in childhood obesity. Biol. Psychol. 92, 199–204. doi: 10.1016/j.biopsycho.2012.09.001, PMID: 22981897

[ref39] Smith J. L. Johnstone S. J. Barry R. J. (2007). Response priming in the Go/Nogo task: the N2 reflects neither inhibition nor conflict. Clin. Neurophysiol. 118, 343–355. doi: 10.1016/j.clinph.2006.09.027, PMID: 17140848

[ref40] Smith E. E. Jonides J. (1999). Storage and executive processes in the frontal lobes. Science 283, 1657–1661. doi: 10.1126/science.283.5408.1657 10073923

[ref41] Song T. F. Chi L. Chu C. H. Chen F. T. Zhou C. Chang Y. K. (2016). Obesity, cardiovascular fitness, and inhibition function: an electrophysiological study. Front. Psychol. 7:1124. doi: 10.3389/fpsyg.2016.01124, PMID: 27512383 PMC4961703

[ref42] Tamm L. Menon V. Reiss A. L. (2002). Maturation of brain function associated with response inhibition. J. Am. Acad. Child Adolesc. Psychiatry 41, 1231–1238. doi: 10.1097/00004583-200210000-00013 12364845

[ref43] Tan S. Y. Guo Y. Y. (2008). Revision of self-control scale for Chinese college students. Chin. J. Clin. Psych. 16, 468–470. doi: 10.3969/j.issn.1006-9771.2004.05.013, PMID: 23020641

[ref44] Tascilar M. E. Turkkahraman D. Oz O. Yucel M. Taskesen M. Eker I.. (2011). P300 auditory event-related potentials in children with obesity: is childhood obesity related to impairment in cognitive functions? Pediatr. Diabetes 12, 589–595. doi: 10.1111/j.1399-5448.2010.00748.x, PMID: 21418454

[ref45] Türkoğlu S. Çetin F. H. (2019). The relationship between chronotype and obesity in children and adolescent with attention deficit hyperactivity disorder. Chronobiol. Int. 36, 1138–1147. doi: 10.1080/07420528.2019.1622131, PMID: 31177853

[ref46] Yang Y. Shields G. S. Guo C. Liu Y. (2018). Executive function performance in obesity and overweight individuals: a meta-analysis and review. Neurosci. Biobehav. Rev. 84, 225–244. doi: 10.1016/j.neubiorev.2017.11.020, PMID: 29203421

[ref47] Yang H. Q. Yao S. Q. Zhu X. Z. Randy P. A. John R. Z. A. Xi T. (2007). The Chinese version of the Barratt impulsiveness scale 11th version(BIS-11) in adolescents: its reliability and validity. Chin. J. Clin. Psych. 15, 4–6. doi: 10.3969/j.issn.1006-9771.2004.05.013, PMID: 23020641

[ref48] Yi X. L. Wang M. Y. Wang X. C. (2015). The relationship between executive functions and pediatric obesity epidemic. Adv. Psychol. Sci. 23, 1920–1930. doi: 10.3724/SP.J.1042.2015.01920

[ref49] Yin J. S. (2016). Event-related potential study of adolescent smoking addiction inhibition control deficit. master’s thesis. Xian: Xidian University.

[ref50] Zelazo P. D. Carlson S. M. (2012). Hot and cool executive function in childhood and adolescence: development and plasticity. Child Dev. Perspect. 6, 354–360. doi: 10.1111/j.1750-8606.2012.00246.x

[ref51] Zhang S. H. (2016). The conflict monitoring of restrained eaters: a single food choice task. master’s thesis. Chongqing: Southwest University.

[ref52] Zhang X. M. Du F. Qian L. J. Yu Q. Wang J. J. Yang P.. (2012). Study of event-related potential P300 in patients with aggressive or violent schizophrenia. Chin. J. Behavior. Med. Brain Sci. 21, 427–429. doi: 10.3760/cma.j.issn.1674-6554.2012.05.013

[ref53] Zhao X. Y. Zheng J. (2001). The origin of event-related potential P300. Chin. J. Neurol. 34, 52–54. doi: 10.3760/j.issn:1006-7876.2001.01.030

